# Immune Checkpoint Inhibitor-Based Systemic Therapy Shows Remarkable Curative Effect in a Hepatocellular Carcinoma Patient With Intractable Postoperative Recurrence and Metastases: A Case Report and Literature Review

**DOI:** 10.3389/fonc.2022.784224

**Published:** 2022-03-15

**Authors:** Xing He, Yaorong Peng, Zhenyu Zhou, Wenbin Li

**Affiliations:** ^1^ Department of Biliary and Pancreatic Surgery, Sun Yat-sen Memorial Hospital, Sun Yat-sen University, Guangzhou, China; ^2^ Department of Hepatobiliary Surgery, Sun Yat-sen Memorial Hospital, Sun Yat-sen University, Guangzhou, China

**Keywords:** hepatocellular carcinoma, metastasis, recurrence, immune checkpoint inhibitor, pembrolizumab, lenvatinib (LEN), TKI (tyrosine kinase inhibitor)

## Abstract

Hepatocellular carcinoma (HCC) is a systemic disease, and most patients make the diagnosis at an advanced stage. In the past, treatments for recurrence of liver cancer with multiple metastases after surgery was very palliative, The case we present is a primary massive HCC patient with inferior vena cava tumor thrombus. Radical hepatectomy was performed in July 2016. Postoperative follow-up showed that sorafenib (a tyrosine kinase inhibitor TKI, 0.8g qd) failed to stop the progression of the disease. Fourteen months later, the patient gradually developed residual liver recurrence, multiple lung metastases and suspected splenic metastasis. The monotherapy regimen was changed from sorafenib to regorafenib (a TKI,160mg qd), but the disease continued to progress. The systematic treatment regimen was changed to Lenvatinib (a TKI, 8mg qd) plus Pembrolizumab (a immune checkpoint inhibitor ICI, 200mg q3w) in April 2019. Following treatment, partial remission (PR) was achieved. According to the mRECIST standard, the PFS has reached 24 months until March 2021, and the overall postoperative survival is 60 months until July 2021. The case we provide show that immune checkpoint inhibitor (ICI)-based systemic therapy may be an effective rescue treatment choice for HCC patients with intractable postoperative recurrence and metastasis.

## Introduction

Hepatocellular carcinoma (HCC) is a common malignant tumor of the digestive system worldwide (1). The situation is even worse in China. The disease is the fourth common malignant tumor and the second leading cause of tumor death in China (2). Due to the lack of screening and follow-up, most of HCC patients are diagnosed at advanced stages and lost the surgery opportunity. Even for early-stage patients treated with surgical resection, the disease recurrence rate is as high as 50%-70% (3, 4). Comprehensive treatment has been demonstrated to benefit for the survival of advanced or recurrent HCC patients (5). IMbrave150 trial (the first ever successful phase III trial of ICIs in advanced HCC) established the combination of atezolizumab plus bevacizumab as the new first-line standard of care for advanced HCC patients, and also certified the potential of ICI-based systemic therapy in HCC (6, 7).

ICI-based systemic therapy includes ICI monotherapy, dual-immunotherapy, ICI plus TKI, ICI plus VEGF inhibitor and ICI plus chemotherapy. Existing studies (7–9) on uHCC have shown that the dual-immunotherapy has higher response rate and better curative effect compared with ICI monotherapy. Combinations incorporating higher doses of anti-CTLA-4 improved efficacy but were also associated with increased anti-CTLA-4 dose-dependent toxicity. Encouragingly, a Phase I/II study (10) demonstrated the most encouraging benefit-risk profile about Tremelimumab plus Durvalumab. For the ICI plus TKI, it has been incorporated into the first-line treatment of several solid tumors (11, 12), Given that several strong biological rationale supports the assessment of ICIs plus TKIs in uHCC (13–16), ICI plus TKI has the potential to represent a novel therapeutic option for uHCC. Notably, no adjuvant treatment is currently recommended in major treatment guidelines, with negative results in various clinical trials and studies (17).

However, there remains an urgent requirement for effective and safe therapeutic agents for the treatment of relapsed *and* refractory HCC, and ICI-based systemic therapies could be an effective strategy. Further studies and clinical cases are necessary to substantiate this perspective (18, 19).

We herein report an advanced HCC patient with inferior vena cava tumor thrombus who failed to respond to multiple postoperative therapies, but achieved partial response soon after ICI combined with TKI. This case may provide more evidence to support that the ICI plus TKI may be a good choice as rescue therapy for HCC postoperative metastatic patients who progress quickly and are resistant to multiple therapies.

## Case Description

**Figure 1:**
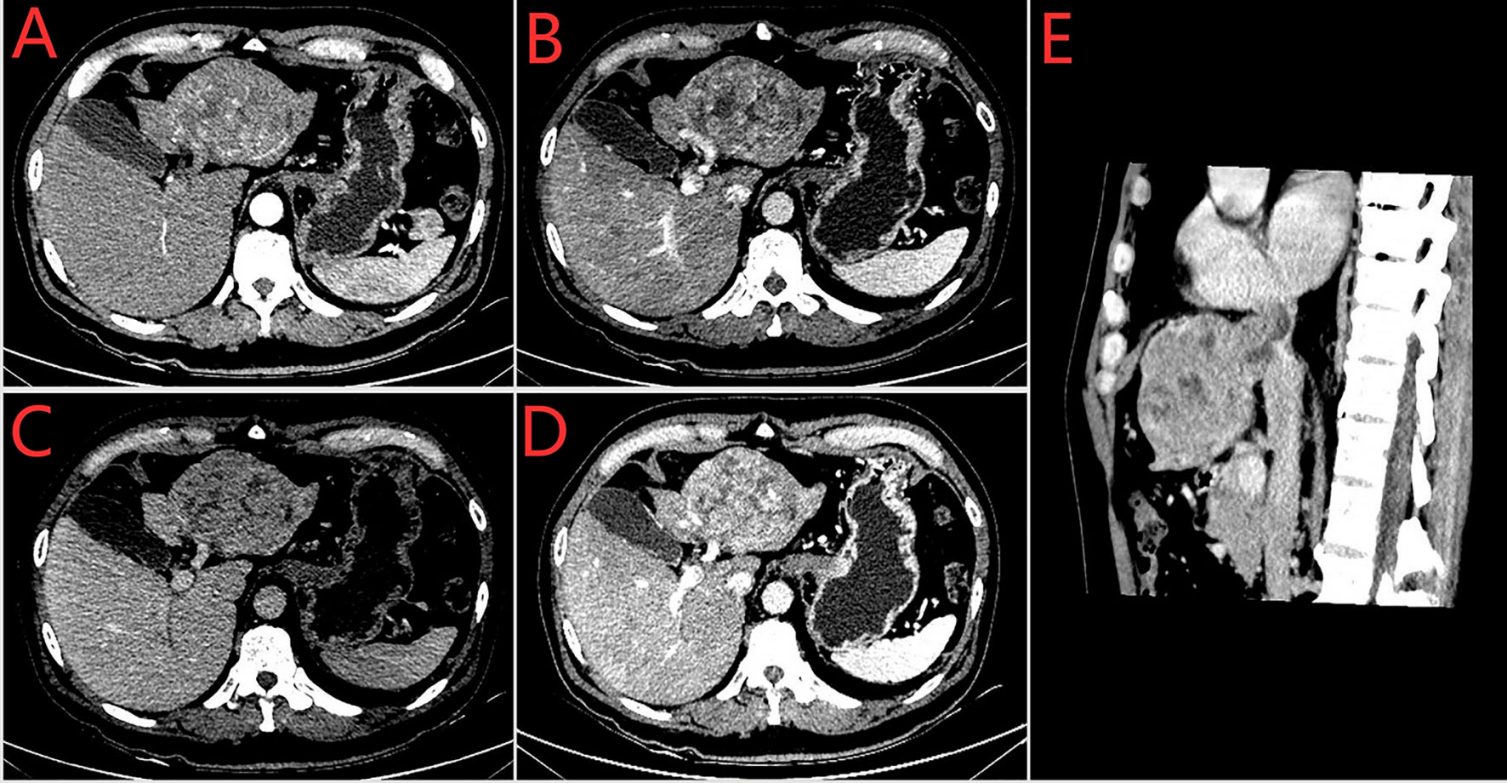
Preoperative enhances-CT of the abdomen (2016–7–7). **(A)** arterial phase **(B)** venous phase **(C)** delayed phase **(D)** The left branch of portal vein was invaded **(E)** Sagital plane; Tumor thrombus of vena cava.

**Figure 2:**
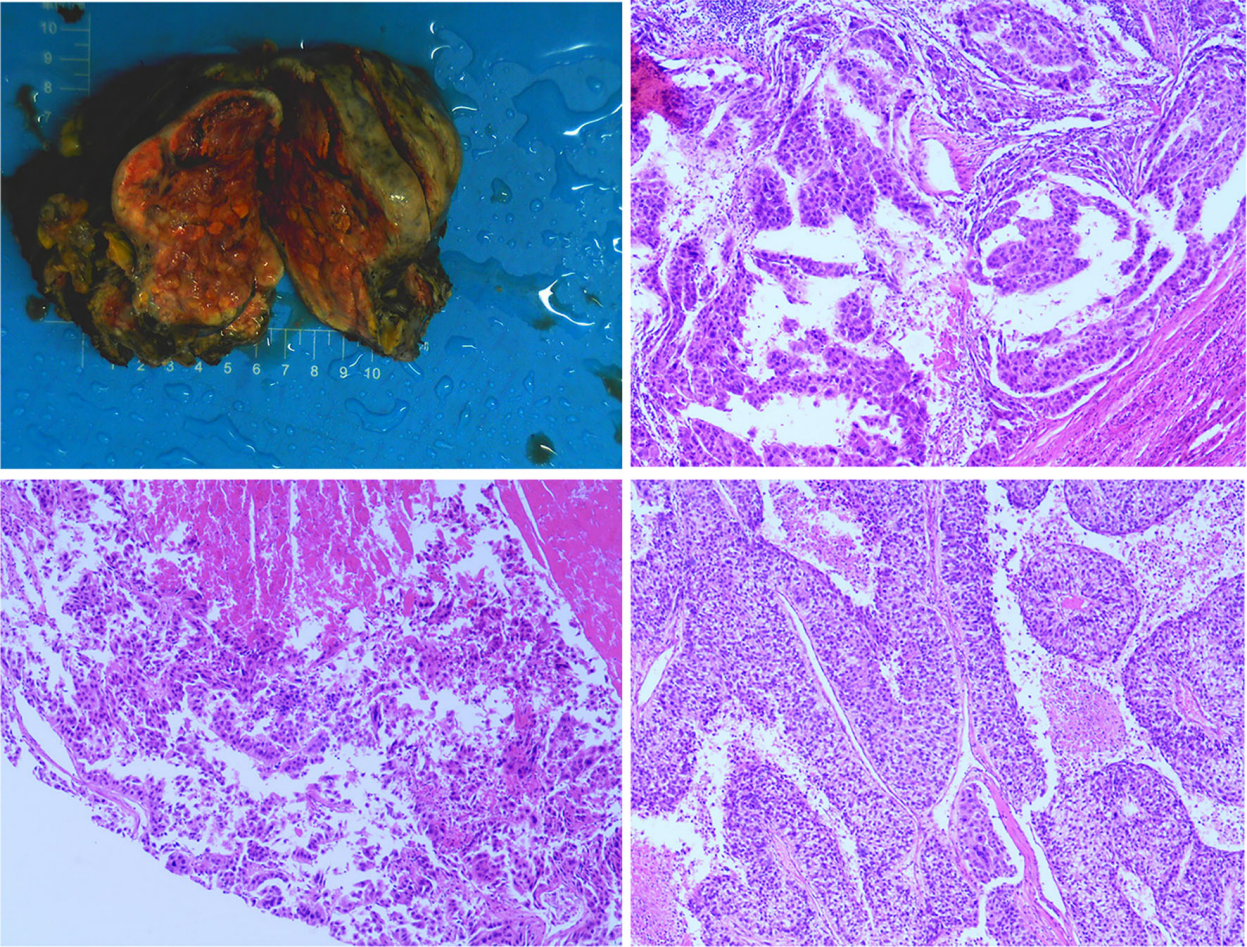
The postoperative specimen was consistent with hepatocellular carcinoma, invaded onto the liver capsule, and a small amount of inflammatory cells infiltrated in the portal area of the surrounding liver tissue. The thrombus removed from the inferior vena cava was consistent with the tumor thrombus.

**Figure 3:**
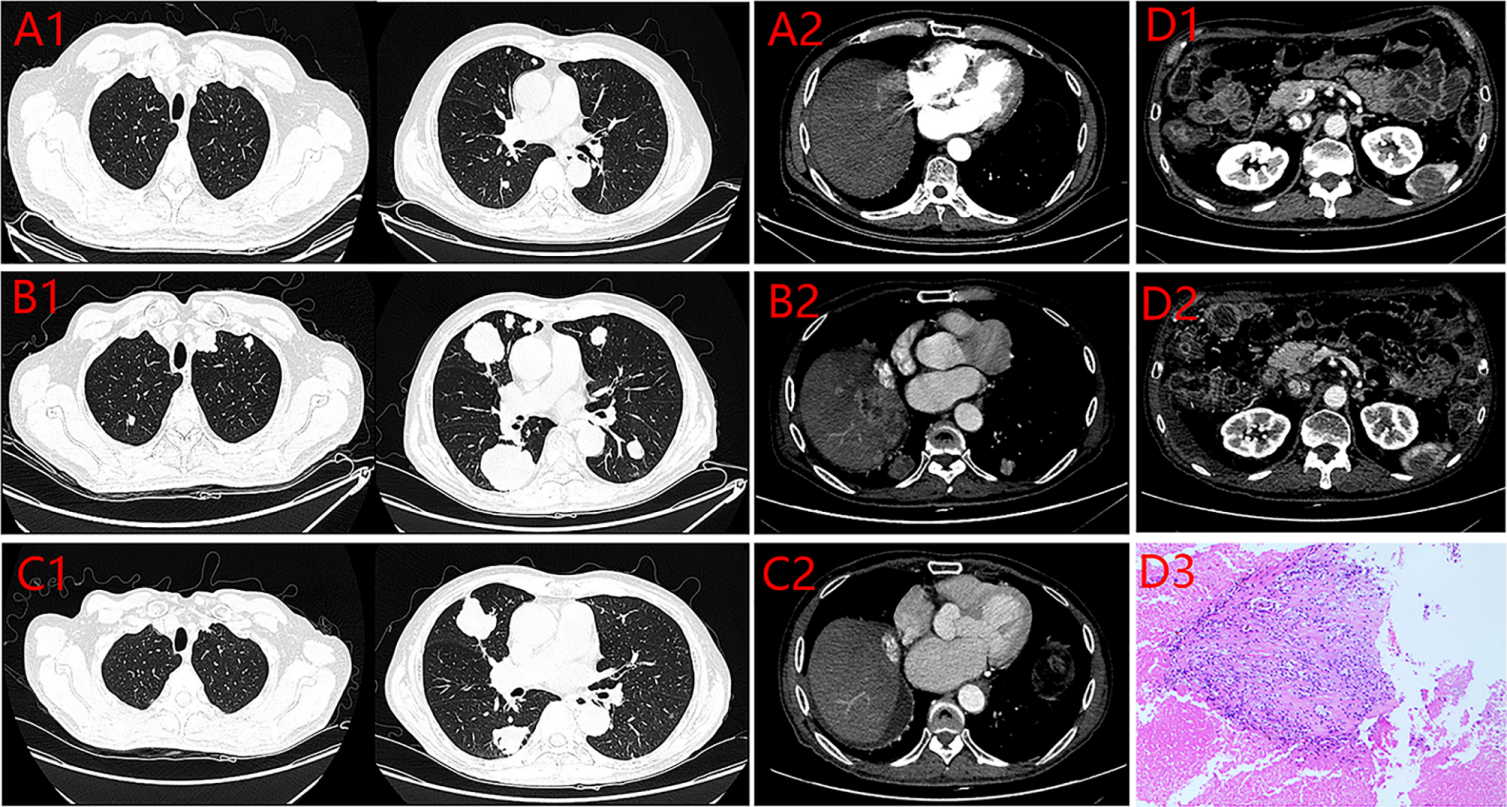
Enhanced CT of the lung and the abdomen. **(A)** 2017.09.17 (1. the Metastatic tumors of Lung 2. the Recurrent focus of Liver). **(B)** 2019.04.19 (1. The Metastatic tumors of Lung 2. the Recurrent focus of Liver). **(C)** 2019.11.11 (1. the Metastatic tumors of Lung 2. the Recurrent focus of Liver). **(D)** 1.spleen lesion (2019.4.19) 2. Spleen lesion after Pem plus Len (2019.11.11) 3. Postoperative pathology of spleen lesion.

**Figure 4:**
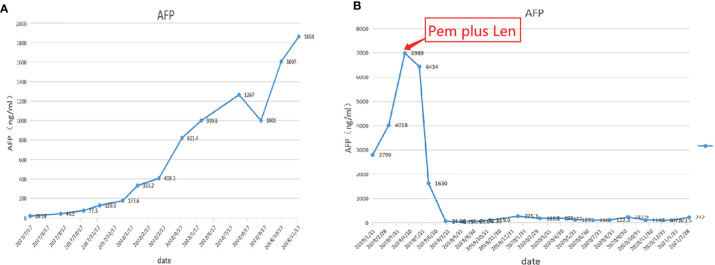
**(A)** AFP level (2017/7/17 ˜ 2018/11/17). **(B)** AFP level (2019/1/31 ˜ 2021/2/28).

A 60-year-old man with a 10-year history of chronic hepatitis B disease and liver cirrhosis presented at hospital with onset of upper abdominal pain in June 2016. Laboratory findings: AFP 43.26ng/ml, HBsAg (+); HBeAb (+); HBVpreS1Ag (+); HBV DNA quantity: 1.65 × 10 ^ 6 copies/ml. Abdominal CT revealed a mass in the left lobe of the liver, and the size of the mass was about 88mm × 79mm; Contrast-enhanced CT showed that the left hepatic vein and the left branch of the portal vein were invaded and a tumor thrombus in the inferior vena cava **(** **Figure 1** **)**, The patient was diagnosed with a primary liver cancer, clinically staged as BCLC C/T4N0M0 lllB/CNLC llla. The patient’s ECOG PS score was 0, the Child-Pugh grade was A (score 5), and the ICG 15R was 15.8%. On the premise of ensuring the residual liver volume more than 40% of standard liver volume, the radical resection was performed under laparotomy in July 2016. During the surgery, the tumor thrombus of the left hepatic vein and inferior vena cava were identified by transesophageal ultrasound and were removed respectively. Postoperative pathology showed that the tumor was R0 resected, and the specimens of the left hepatic vein and inferior vena cava were confirmed as tumor thrombus **(** **Figure 2** **)**. The patient began to receive chemotherapy (SOX:S1 60mg bid d1-d14, Calcium levofolinate 200mg q3w, Oxaliplatin 150mg q3w) plus Sorafenib (400mg bid) after the operation, supplemented with anti-hepatitis B virus therapy, liver protection therapy, TACE was also carried out two times(August 2016; February 2017);. The dosage of sorafenib was reduced to 400mg each morning and 200mg each night due to hand-foot adverse reaction, The patient recovered well post-operatively and no clinical or radiological evidence of recurrence were noted at the follow-up. Unfortunately, 14 months after the surgery (September 2017), the AFP level showed an upward trend **(** **Figure 4A** **)**, and contrast-enhanced CT showed recurrence of liver cancer and multiple lung metastases **(** **Figure 3A** **)**. The patient underwent TACE twice in November 2017 and December 2017 respectively, but this did not reverse the progression of the disease. The targeted therapy plan was changed to Regorafenib 60mg once a day(D1-D21 each month)in March 2018. However, the AFP level still showed an upward trend. Contrast-enhanced CT scan showed that lesions in liver and lung were enlarged, and a suspicious lesion in the spleen was found in April 2019 **(** **Figure 3D1** **)**. The disease was in a progress-disease (PD) status according to the mRECIST criteria. The systematic treatment regimen was changed to Lenvatinib (8mg qd) plus Pembrolizumab (an immune checkpoint inhibitor ICI, 200mg q3w) in April 2019. (100mg q3w, did not use 200mg considering the overall status of the patients. the Child-Pugh grade was B) in April 2019. Later, the dose of Pembrolizumab was increased to 200mg q3w because of the patient’s well tolerance. After two courses of treatments, the AFP level decreased significantly **(** **Figure 4B** **)**, and CT scan also showed significant reduction of the recurrent liver lesions and pulmonary metastases **(** **Figures 3B, C** **)**. Following treatment, partial remission was achieved. The active lesions of liver recurrence disappeared completely, which leave low-density central necrosis **(** **Figure 3C2** **)**. The suspicious metastatic tumor in the spleen also shrank significantly **(** **Figure 3D2** **)**. Laparoscopic splenectomy was performed in November 2019. The postoperative pathology showed no residual cancer cell was found in the extensive nodular necrosis in the spleen, which is consistent with the changes after systemic therapy **(** **Figure 3D3** **)**. The patient has continued this systematic treatment regimen so far, the general condition is well, and the liver function continues to maintain grade A. According to the mRECIST standard, the PFS has reached 24 months until March 2021 **(**Chest lesions after November 2020 were evaluated by chest X-rays, which showed that the pulmonary lesions had enlarged since March 2021, presenting PD status**)**. and the overall postoperative survival is 60 months until July 2021.

## Discussion

In the past, HCC patients with inferior vena cava tumor thrombus had significantly shorter survival, even after radical resection. How to maximally prolong postoperative overall survival has always been one of the top priorities for surgeons. We searched the Pubmed database and found that we report the longest overall survival of HCC patients with inferior vena cava tumor thrombus and refractory recurrence after surgery. After the failure of sorafenib and regorafenib, the patient was treated with Lenvatinib plus Pembrolizumab and achieved a PFS of 24 months. Combination therapy is well tolerated. Additionally, despite the promising improvement that the combination of atezolizumab and bevacizumab showed as a first-line option, the exclusion criteria include more advanced uncompensated cirrhosis beyond Child-Pugh A class. Further progress may be particularly difficult due to the challenges of treating specific patient populations with underlying uncompensated cirrhosis. Our case provides limited evidence for the effectiveness of TKI plus ICI in treating HCC patients with uncompensated cirrhosis. In China, TKI is an attractive choice for HCC patients as TKIs are convenient to take and require less frequent hospital visits and medical costs. Thus, TKI plus ICI are able to bring better patient adherence and drug tolerance when the disease progressed. Of course, our case has some limitations. we cannot exclude that the patient would also have responded to Lenvatinib monotherapy. However, our case are still meaningful because it provides limited evidence that the ICI plus TKI may be a good choice as rescue therapy for HCC postoperative metastatic patients who progress quickly and are resistant to multiple therapies.

Reviewing the treatment of recurrent hepatocellular carcinoma, the situation is not optimistic (3, 4). How to effectively treat postoperative recurrence and further prolong the overall survival time of patients has always been a problem. There are basically no large-scale clinical studies for the treatment of recurrence after hepatectomy, and some existing clinical evidences are aimed at the recurrence after liver transplantation (20–22). ICI therapy in patients with a history of liver transplantation needs to be considered with a great deal of caution given the possibility of graft rejection. However, recurrent HCC after hepatectomy will not be bound by this. With the deepening understanding of the immune microenvironment of HCC, immunotherapy using the immune mechanism of the body to enhance tumor immune response and block tumor immunosuppression has become a new direction for the treatment of HCC, among which the immune checkpoint inhibitors (ICIs) have been most widely used (23). However, contrary to melanoma and non-small cell lung cancer, the response rate to ICI treatments in HCC remains low (24), the situation indicate that it may be necessary to combine ICI with other drugs to improve the efficacy. As a vascular rich tumor, the special structure of the new vessel wall of HCC often makes it difficult for anti-tumor drugs and immune cells to reach the tumor site. Therefore, strategies aiming at combining antiangiogenic drugs related signaling with ICI therapy could be the ideal regimen to further overcome the immunosuppressive nature of the TME by inducing tumor vascular normalization as well as enhancing DC maturation, optimizing the treatment efficacy of ICI therapy. Lenvatinib(LEN), a representative oral multi-kinase inhibitor, is able to target fibroblast growth factor receptor, vascular endothelial growth factor receptor, RET, KIT, and other kinases. Numerous studies have provided evidence to support the combination of LEN and ICI. In preclinical murine models, the combination of LEN with the anti-PD-1 antibody has been shown to enhance antitumor activity (25). LEN significantly decreased the population of tumor-associated macrophages, as well as increased the percentage of activated CD8+ T cells secreting interferon-g+ and granzyme B. In addition, LEN significantly reduced the level of tumor programmed death ligand 1 (PD-L1) and Treg differentiation, improved anti-PD-1 efficacy by blocking FGFR4, and inhibiting TGF-ß signaling (16, 24, 26). Supported by above findings, several studies are ongoing. KEYNOTE-524/Study116 (27), an open, one-arm phase Ib clinical study, aims to evaluate the efficacy and safety of Pembrolizumab combined with Lenvatinib in first-line treatment of advanced unresectable hepatocellular carcinoma patients who cannot be locally treated. The results show that the median OS is 22. 0 months, the median PFS is 9. 3 months, and the ORR is 46%. Based on these results, the FDA granted breakthrough drug status to pembrolizumab in combination with lenvatinib for thefirst-line treatment of advanced unresectable HCC in July 2019. A phase I clinical trial (n=18) (28) first evaluated the effect of Camrelizumab (an ICI) combined with Apatinib(a TKI) in the treatment of HCC. The results showed that ORR was 50.0%, DCR was 93.8% and the mPFs was 5.8 months. At the same time, a non -randomized, open, multi-center phase II clinical trial RESCUE (29) was launched to evaluate the effectiveness and safety of combination therapies in the first-line and second-line treatment of HCC. The results showed that the ORR of the first-line and the second-line treatment group was 34.3% and 22.5%, respectively. The mPFS of the two groups were 5.7 and 5.5 months, and the corresponding 12-month OS was 74.7% and 68.2%, respectively. A real-world retrospective study showed that Lenvatinib plus ICIs provided significantly higher overall survival (hazard ratio = 0.47, 95% CI 0.26-0.85; p = 0.013) and progression-free survival (hazard ratio = 0.35, 95% CI 0.20-0.63; p < 0.001) than lenvatinib monotherapy (30). At present, an international, multicenter, phase III clinical study (LEAP-002) of pembrolizumab +lenvatinib for uHCC is ongoing. The results will be published in 2021. Other clinical trials regarding the combination of ICIs with TKI are underway, including trials of nivolumab + cabozantinib (NCT01658878, NCT03299946) and nivolumab + lenvatinib (NCT03418922, NCT03841201) for advanced HCC; findings from these trials are also eagerly expected.

Therapy of HCC is still unsatisfactory despite the fact that the advent of ICIs has led to tremendous progress. There is still much room for immunotherapy studies. Firstly, the mechanisms of different combination therapies should be illustrated, and new agents evaluated. Better understanding may pave the way to more optimized combination with different ICIs (e.g., certain TKIs may benefit from the addition of CTLA-4 whilst others may only benefit from anti-PD-1/L1 combinations.) Secondly, blocking the compensatory upregulation of other checkpoint inhibitors such as TIM3 and LAG3 after anti-PD1/PDL1 treatment could be an important pharmaceutical strategy to overcome primary and secondary resistance to PD1/PDL1 blockade in patients with advanced HCC in the future. Further preclinical and clinical studies will advance this important and promising area of research. Thirdly, we still need to work on finding biomarkers that can suggest a good response to ICIs, which have certain guiding significance for distinguishing the beneficiaries of ICIs treatment and evaluating the efficacy of immunotherapy. It might help advance personalized therapy. Lastly, the majority of HCC patients have concomitant cirrhosis, data on the use of ICI and ICI combinations in such patients is deficient. Efforts should be made to embed correlative screening criteria in some new clinical trial or prospective study.

## Data Availability Statement

The original contributions presented in the study are included in the article/**Supplementary Material**. Further inquiries can be directed to the corresponding author.

## Ethics Statement

The studies involving human participants were reviewed and approved by the Ethics Committees of Sun Yat-sen Memorial Hospital, Sun Yat-sen University. The patients/participants provided their written informed consent to participate in this study.

## Author Contributions

XH and WL carried out the studies, participated in collecting data, and drafted the manuscript. YP and ZZ revised and commented on the draft. All authors contributed to the article and approved the submitted version.

## Funding

National Natural Science Foundation of China (Grant no. 81301768); Science and Technology Program of Guangdong Province, China (No.2018A030313809, 2021A1515010109).

## Conflict of Interest

The authors declare that the research was conducted in the absence of any commercial or financial relationships that could be construed as a potential conflict of interest.

## Publisher’s Note

All claims expressed in this article are solely those of the authors and do not necessarily represent those of their affiliated organizations, or those of the publisher, the editors and the reviewers. Any product that may be evaluated in this article, or claim that may be made by its manufacturer, is not guaranteed or endorsed by the publisher.
